# Knowledge, Attitude, and Practices on Antimicrobial Use and Antimicrobial Resistance among Commercial Poultry Farmers in Bangladesh

**DOI:** 10.3390/antibiotics10070784

**Published:** 2021-06-28

**Authors:** Mohammad Mahmudul Hassan, Md. Abul Kalam, Md. Abdul Alim, Shahanaj Shano, Md. Raihan Khan Nayem, Md. Rahim Badsha, Md. Abdullah Al Mamun, Ashraful Hoque, Abu Zubayer Tanzin, Chandan Nath, Hamida Khanom, Shahneaz Ali Khan, Md. Mazharul Islam, Md Bashir Uddin, Ariful Islam

**Affiliations:** 1 Faculty of Veterinary Medicine, Chattogram Veterinary and Animal Sciences University, Chattogram 4225, Bangladesh; maalim85@gmail.com (M.A.A.); raihannayem@gmail.com (M.R.K.N.); mabdul-lahal8@gmail.com (M.A.A.M.); ashraful.cvasu@gmail.com (A.H.); tanjin04@gmail.com (A.Z.T.); chandannath227@gmail.com (C.N.); hamidakhanom21@gmail.com (H.K.); shahneaz-bat@gmail.com (S.A.K.); 2 Helen Keller International, Dhaka 1212, Bangladesh; a.kalam724@gmail.com; 3 EcoHealth Alliance, New York, NY 10001-2320, USA; shahanajshano@gmail.com (S.S.); arif@ecohealthalliance.org (A.I.); 4 Institute of Epidemiology, Disease Control and Research (IEDCR), Dhaka 1212, Bangladesh; 5 Faculty of Food Science and Technology, Chattogram Veterinary and Animal Sciences University, Chattogram 4225, Bangladesh; mrbadsha1322@gmail.com; 6 Department of Animal Resources, Ministry of Municipality and Environment, Doha P.O. Box 35081, Qatar; mmmohammed@mme.gov.qa; 7 Department of Medicine, Sylhet Agricultural University, Sylhet 3100, Bangladesh; 8 Centre for Integrative Ecology, School of Life and Environmental Science, Deakin University, Geelong Campus, Warrnambool, VIC 3216, Australia

**Keywords:** food safety, poultry farms, poultry farmers, antimicrobial use, antimicrobial resistance, KAP, Bangladesh

## Abstract

Antimicrobial resistance (AMR) has become an emerging health issue globally, posing a threat to zoonotic pathogens and foodborne diseases. In Bangladesh, the poultry sector supplies the majority of the demand for animal-source protein. The irrational and excessive use of antimicrobials (AMU) has been observed in the poultry sector. The development of AMR is associated with many factors, including the knowledge and attitudes of poultry farmers. Therefore, AMR reduction requires intervention from all the stockholders, including the farmers who are considered as end users of antimicrobials. This current research conducted a cross-sectional study to assess the knowledge, attitudes, and practices (KAP) of poultry farmers on AMU and AMR in Bangladesh. We determined the KAP of poultry farmers (broiler and layer farmers) of some selected districts of the country using a tested and paper-based questionnaire. The results demonstrated that most of the respondents have insufficient KAP regarding AMU and AMR. The respondents used a variety of antimicrobials primarily in the treatment of various diseases in poultry. One-third of the farmers did not seek antimicrobials from registered vets. Instead, they depended on others or themselves. The factor score analysis further revealed that the farmers’ demographic and socioeconomic variables were significant factors influencing the KAP. An adjusted logistic regression analysis showed that older farmers with 9–12 years of farming experience and graduate-level education, engaging in medium-sized layer farming, were more likely to have correct KAP on AMU and AMR. Further, farmers from the Cox’s Bazar region showed correct knowledge, whereas farmers of the Chattogram region showed a correct attitude towards AMU and AMR. A Spearman’s rank-order correlation revealed a positive association between knowledge–attitudes and knowledge–practices. The findings of the current investigation provide baseline evidence about the KAP of poultry farmers from low-income resources and offer insights into designing interventions and policies for the use of AMU and AMR in Bangladesh.

## 1. Introduction

Livestock production has seen a massive expansion as a result of intensive farming systems in order to meet the demand for animal-origin proteins for the growing population through efficient and economically sustainable methods. In Bangladesh, poultry farming is becoming a sustainable resource for generating economic growth in rural communities. According to the Bangladesh livestock economy of 2019 to 2020, an amount of 3563.18 lakh of poultry was produced from 4122.44 lakh of the total livestock production [1]. By supplying animal-source proteins for humans, poultry industry plays a significant role in the economy of Bangladesh through providing employment opportunities and contributions to the country’s Gross Domestic Product [1]. Moreover, poultry has supplied a substantial amount of meat and eggs throughout the years to meet protein demands. This phenomenon put pressure on commercial poultry farmers to produce more meat and eggs for the growing population and triggered the unregulated use of growth promoters and probiotics to meet the high production demands [2]. Antimicrobial use (AMU) in food-producing animals has benefited by improving the health, productivity, and economic returns by reducing the disease incidence, morbidity, and mortality from the therapeutic levels, while the growth-promoting and enhancing feed efficiency are considered in the context of nontherapeutic purposes [3]. Using antimicrobials in poultry production systems increases the chance of antimicrobial resistance (AMR). Antimicrobials are essential to protect animal health in livestock production systems, but their misuse creates a favorable niche for AMR bacteria in livestock farms, wildlife, and the environment, which might transmit to humans through contaminated foods or direct contact [4,5,6,7,8,9,10]. Humans consuming livestock and aquaculture-derived food products might develop resistance against specific antimicrobials due to antimicrobial residues being introduced into the human food chain [11,12,13,14,15,16,17]. They could pose a threat to the human population through increasing exposure to zoonotic pathogens and foodborne diseases [18]. Recent research has raised concerns about the increased exposure of antimicrobials use and antimicrobial resistance in Bangladesh, which governs public health concerns [19,20].

Nevertheless, AMR has increased rapidly in the last decade and has become a global threat, as it becomes increasingly difficult to treat and control infectious diseases both in humans and animals [21]. The lack of legislation, regulations on using drugs, and their self-prescription by farmers as the highest level of medication in livestock are responsible for developing AMR, which is a significant threat to public health worldwide [22]. Like many other countries, the government of Bangladesh enacted the National Action Plan (NAP) on AMR in 2017 [23]; however, the plan has focused more on surveillance rather than the inclusion of multi-stakeholders to address the AMR problem. Poultry farmers are key risk factors in the veterinary drug chain, and most of them are not well-trained in poultry farming. Thus, they might contribute to the incidence and spread of AMR through the misuse and indiscriminate use of antibiotics.

Therefore, it has become crucial to control antibiotics, monitor resistance, and develop new strategies to reduce antimicrobial resistance in poultry farms. One key strategy is to increase the knowledge and skills of the community and farmers about judicial antibiotic use and build favorable attitudes towards AMU through awareness campaigns. It is of utmost importance to assess the present scenario in Bangladesh to develop and implement effective control measures against AMR. Veterinarians and farmers are the two critical users of antimicrobials for poultry farms. Enforcing the relevant regulations for the farmers is an essential step to mitigating the AMR problem in Bangladesh. One possible way to prevent intensive and unnecessary antimicrobials use in animals is to inform farmers about the antibiotics, residues, and resistance, which may decrease drug resistance in humans. The knowledge and behavior of farmers can significantly influence their decision to AMU [24,25,26]. However, the current antimicrobial stewardship training, curriculums, and guidelines limit the value in selecting antimicrobials and the transmission of AMR [27,28,29]. Better knowledge, attitudes, and practices were associated with producers who engaged in exploring information on AMU and AMR [30]. Therefore, the primary objective of this study was to assess the knowledge, attitude, and practices (KAP) on AMU and AMR among commercial poultry farmers in Bangladesh. The secondary objective of this study was to inform current policy initiatives to control AMR as part of the NAP of Antimicrobial Resistance in Bangladesh and beyond.

## 2. Results

### 2.1. Demographic and Socioeconomic Characteristics of the Respondents

We conducted a total of 420 interviews (Supplementary Table S1). The characteristics of the study interviewees are presented in Table 1 and Supplementary Table S1. Out of the 420 respondents, 210 were broiler and layer farmers, respectively. Equal numbers (*n* = 60) of farmers were recruited from seven districts. All the respondents were male, and most of them belonged to the 18–30 years of age group (*n* = 140). The majority of respondents had 9–12 years of farming experience (*n* = 153), and received education up to the 12th grade (*n* = 308). The vast majority of respondents had poultry farming as their primary income source (*n* = 390), belonged to the middle-income group (*n* = 344), and were involved in small-scale farming (*n* = 262).

### 2.2. Recent Diseases in the Farm, Antimicrobial Seeking, Performing Post-Mortem, and Used Antimicrobials on the Farm

**Figure 1:**
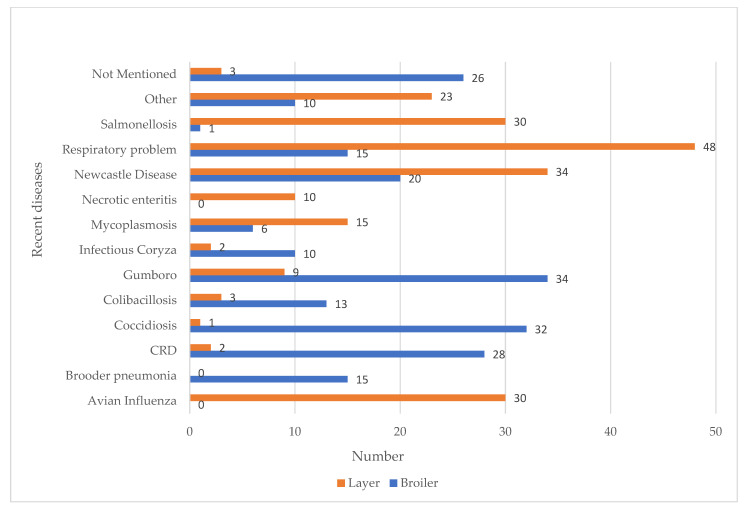
Incidence of diseases on broiler and layer farms.

The primary disease conditions reported by the farmers within the last 6 months on their farms are listed in Figure 1. Among the diseases, respiratory problems or Chronic Respiratory Disease (CRD) (*n* = 48), Newcastle Diseases (*n* = 34), Avian Influenza and Salmonellosis (*n* = 30, respectively) were reported as the top three diseases on layer farms, while Gumboro (*n* = 34), Coccidiosis (*n* = 32), and CRD (*n* = 30) were found on broiler farms.

**Figure 2:**
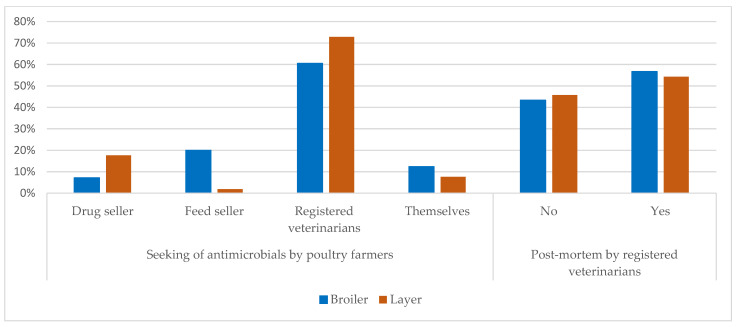
Seeking out antimicrobials by broiler and layer farmers and performing post-mortem examinations before using antimicrobials.

The poultry farmers sought out antimicrobials for diseases and disease conditions (Figure 1) on farms from several sources (Figure 2). The majority of all the farmers (broiler and layer together) (67%) depended on registered veterinarians, considered a good practice. Layer farmers were more likely to seek out antimicrobials from a registered vet compared to broiler farmers (73% and 60%, respectively). However, a good proportion (33%) of all the farmers sought out antimicrobials from different sources other than registered veterinarians. A disaggregated analysis by the type of farmer showed that broiler farmers were more likely to seek out antimicrobials from a feed seller, while layer farmers preferred to go to drug sellers to seek antimicrobials. The broiler farmers tended to seek antimicrobials by themselves compared to layer farmers. Before choosing an appropriate antimicrobial, about 45% of the broiler and layer farmers reported not performing a post-mortem examination by a veterinarian in order to identify the diseases.

**Figure 3:**
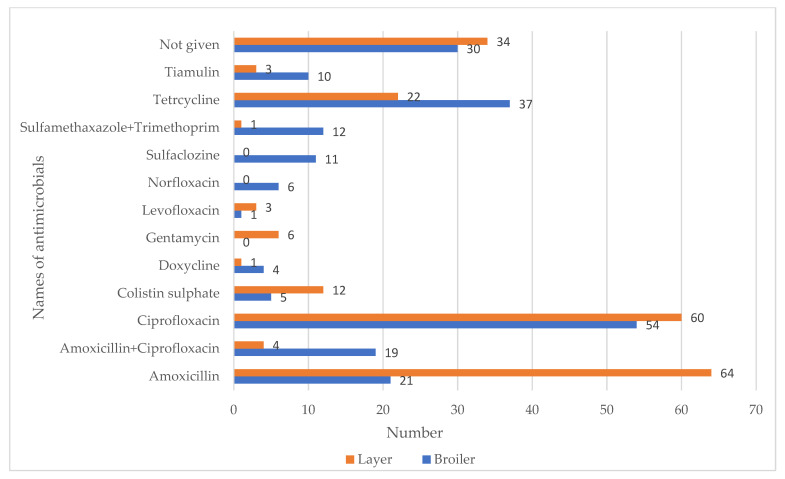
Use of antimicrobials on poultry farms against different diseases (disaggregated by the type of farm).

As shown in Figure 3, the poultry farmers reported that they used several antimicrobials against recent diseases on their farms. Amoxicillin (*n* = 64) was the most commonly used antimicrobial, followed by ciprofloxacin (*n* = 60) and tetracycline (*n* = 22), on layer farms. On the other hand, ciprofloxacin (*n* = 54) was the most frequently used antimicrobial, followed by tetracycline (*n* = 37) and amoxicillin (*n* = 21), on broiler farms. Interestingly, a considerable number of farmers (*n* = 34 and 30 from layer and broiler farms, respectively) said no antimicrobials were applied on their farms.

### 2.3. Knowledge

We asked twelve questions, nine positive and three negative, to assess the farmers’ knowledge of AMU and AMR. The results are presented in Table 2. In general, the farmers’ self-reported responses showed that most of them had a familiarity with the authority of prescribing antimicrobials (*n* = 360). However, considering the item-based questions, the proportion of desirable answers and significance level (as a means of the chi-square test) were higher in layer farmers than in broiler farmers.

Additionally, we asked farmers if they were familiar with terms such as “Antimicrobials”, “Antimicrobial resistance”, and “Antimicrobial residue”. The analysis showed that the vast majority of the respondents (92.1%) had an idea of what antimicrobials are, while more layer farmers were aware of antimicrobials than broiler farmers (96.2% and 88.1%, respectively; *p* = 0.002). However, while asking about antimicrobial residues and antimicrobial resistance, the proportion of correct responses was 63.3% and 56.7%, respectively. In both cases, the desirable responses were found significantly higher among layer farmers compare to broiler farmers.

The responses to the question “Do you know any specific antimicrobials that act against a specific disease?” showed reasonably good knowledge (84.5% of the total respondents said “Yes”). However, the broiler farmers were significantly mentioned this response. Similarly, most of the farmers (80.5% of the total farmers) said that “…antimicrobials can be passed to humans through consumption of poultry meat and egg”, showing a good knowledge of animal transmission of AMR to humans. The vast majority of the farmers (97.1%) said “Yes” to “Do you think antimicrobials have some side effects?”, showing correct knowledge, and this response was significantly higher in layer farmers compared to broiler farmers (99.5% and 94.7%, respectively; *p* = 0.003). The majority of the farmers (91.2%) said “Yes” to “Do you think the treatment is needed for the whole flock if one/few birds show any symptoms?”, and approximately the same number of broiler and layer farmers (190 and 193, respectively) mentioned this response.

Regarding the negative items, overall, the desirable responses were pretty good among the respondents. Specifically, 71.1% of total farmers said “No” to “Did you know antimicrobials can be used for any disease?”, around 60% said “No” to “Do you think antimicrobials are efficient for the treatment of both bacterial and viral infections?”, and almost 80% said “No” to “Do you think all antimicrobials can show the same curative effect in poultry diseases?”. However, the comparative analysis showed significantly more layer farmers said “No” to the former two items (*p* = 0.000 and *p* = 0.011, respectively) compared to broiler farmers. On the contrary, broiler farmers said “No” in response to the latter items (*p* = 0.000) significantly more often than layer farmers.

### 2.4. Attitudes on AMU and AMR of Broiler and Layer Farmers

We asked nine questions, five positive and four negative, to assess farmers’ attitudes towards AMU and AMR on broiler and layer farms. Overall, the pattern of desirable responses to the attitude questions revealed a similarity to the knowledge responses (as reported in the section above), in that, layer farmers gave more correct responses than broiler farmers (Table 3).

The majority of the farmers (62.9% of the total respondents) answered “No” to the statement “Do you think antimicrobials should be added to feed at any time to prevent birds from becoming sick?”, which is a correct response. This response was given significantly more by layer farmers. Approximately the same proportion (62.2%) of the total farmers (*n* = 420) believed that both the “Random use of antimicrobials” and “Missing a dose of antimicrobials” may contribute to the development of AMR.

A general attitude observed was that most farmers believed that antimicrobials should be placed in a restricted place and accessed by the farmers or a specific person: only 18.1% of all the respondents mentioned the desirable response “No”. Similarly, little more than half of the respondents (56.4%) reported “Yes” to “restriction on antimicrobial use can cause more damage than benefit”, representing an appropriate attitude.

Eighty-eight percent of respondents felt the importance of accurate doses of antimicrobials in controlling inappropriate antimicrobials in the poultry sector. This response was significantly higher in layer farmers compared to broiler farmers. The vast majority of the respondent said “No” to the question “When antimicrobials are about to expire, is it better to give medication to the birds to prevent wastage?”, depicting a positive attitude. This response was significantly more common in broiler farmers than layer farmers.

Most farmers (71.7%) thought that herbals or medicinal drugs could be used as alternatives to antimicrobials. This response was significantly higher in layer farmers compared to broiler farmers. Similarly, the vast majority of the farmers (80.5%) thought that having accurate information on random uses may reduce antimicrobials in the future. This response was also higher among layer farmers compared to broiler farmers.

### 2.5. Practices towards AMU and AMR in Broiler and Layer Farmers

We asked eleven questions, three positive and eight negative, to assess farmers’ practices regarding AMU and AMR. The results are shown in Table 4. Some practices considered to be at risk for AMR were common between both groups. Self-medication was prevalent in similar proportions among layer and broiler farmers. A mentionable proportion (30.2% of all the farmers) of the respondents reported they had used antimicrobials by themselves, and this response was found to be significantly higher among layer farmers compared to broiler farmers (41.4% vs. 19.1%, *p* = 0.000). In terms of using antimicrobials during the brooding period, only 18.1% of all farmers reported the desirable response “No”, which means the vast majority of the farmers (81.9%) followed this practice inappropriately. When asked, “Do you seek suggestions for using antimicrobials from a non-vet?”, only 20.5% of the farmers said “No”—meaning the majority of them practiced as such. This practice was observed significantly more among layer farmers compared to broiler farmers.

Conversely, most of the farmers (94.1%) checked the expired date before purchasing antimicrobials, which is a good practice. This practice was found significantly more common among layer farmers than broiler farmers (97.6% vs. 90.5%, respectively; *p* = 0.002).

More than half of the farmers (56.7%) reported “No” to being asked, “Do you use antimicrobials as a growth promoter?”, which indicated that a good proportion of the farmers used antimicrobials as a growth promoter, a misuse of antimicrobials. A little less than 50% of all the respondents reported seeking advice from veterinarians about the withdrawal period, and this response was significantly more common among layer farmers.

Layer farmers significantly reported “No” while asking, “Do you increase in the antimicrobial dose and frequency when there is no sign of recovery?” (65.2% and 45.7%, respectively), representing a good practice. Similarly, a more significant proportion of farmers (71.2% of the total farmers) responded “No” to the negative statement, “Do you shift to using different antimicrobials during the course of a disease?”—representing an appropriate practice. This response was found to be significantly more common among layer farmers than broiler farmers (78.1% and 65.3%, respectively; *p* = 0.002). Same as the two negative statements above, the majority of the farmers said “No” when asked, “Do you eat the meat of birds that are given antimicrobials at the end state?”, which depicted an excellent practice. This response was found almost equally among the broiler and layer farmers.

### 2.6. Differences in Knowledge, Attitudes, and Practices on AMU and AMR in Broiler and Layer Farmers

A principal factor analysis was performed to show the significant characteristics between the demographic variables and knowledge themes. The results are given in Table 5: the age of respondents (*p* = 0.000), years of experiences in farming (*p* = 0.000), economic status of the respondents (*p* = 0.001), level of education (*p* = 0.000), farm size (*p* = 0.013), and farm type (*p* = 0.000) were significant factors influencing their knowledge and practices. The analysis revealed that the geographic location influences the correct knowledge (*p* = 0.005) of AMU and AMR.

The analysis further revealed that the respondents’ age (*p* = 0.000), years of experience in farming (*p* = 0.000), economic status (*p* = 0.001), level of formal education (*p* = 0.007), and farm size (*p* = 0.000) were significant factors affecting their attitudes.

In terms of respondents’ practices on AMU according to their demographic and socioeconomic variables, the principal factor analysis demonstrated that the key factors were the respondents’ age (*p* = 0.000), years of experiences in farming (*p* = 0.000), economic status of the respondents (*p* = 0.000), level of education (*p* = 0.000), farm size (*p* = 0.013), and farm type (*p* = 0.005). Geographic variations were also found to have a significant influence on practices (*p* = 0.000) on AMU and AMR.

The output of the adjusted logistic regression analysis of the respondents’ demographic variables and their levels of knowledge, attitudes, and practices are presented in Table 6. The results showed that respondents’ ages were positively associated with the increased levels of knowledge on AMU and AMR. Specifically, the farmers between 36 and 40 years of age had 0.13 times, those who were between 41 and 45 years of age had 0.17 times, and those who were 46 or older had 0.23 times the correct knowledge on AMU and AMR (OR = 0.13, CI = 0.05–0.34; *p* = 0.00; OR = 0.17, CI = 0.07–0.39, *p* = 0.000; and OR = 0.23, CI = 0.10–0.52, *p* = 0.000, respectively), compared to the farmers who were between 18–35 years of age.

The results revealed that the respondents’ experiences in poultry farming correlated closely to the level of correct knowledge. Precisely, the farmers who had experiences between 5 and 8 years had 7.13 times, between 9 and 12 years had 11.54 times, and more than 13 years had 7.27 times the correct knowledge of AMU and AMR compared to those farmers who had less than 4 years of experience (OR = 7.13, CI = 3.16–16.06, *p* = 0.000; OR = 11.54, CI = 4.84–27.51, *p* = 0.000; and OR = 7.27, CI = 2.46–21.45, *p* = 0.000, respectively).

Similarly, the outcomes revealed that the level of education was positively correlated with the increased levels of knowledge. The farmers who completed graduation had 2.96 times the “correct” knowledge than farmers who received education up to the 12th grade (OR = 2.99, CI = 1.69–5.20, *p* = 0.000). Further, the farmers who raised layer poultry had 2.01 times the “correct” knowledge on AMU and AMR compared to their counterparts, broiler farmers (OR = 2.01, CI = 1.19–3.39, *p* = 0.009). In addition, the farm size was a significant association with farmers’ levels of knowledge. The farmers who owned medium-sized farms had 3.95 times the correct knowledge than those who owned small-sized farms (OR = 3.95, CI = 2.08–7.50, *p* = 0.000). The Cox’s Bazar district was one of the best-performing geographic areas in terms of knowledge on AMU and AMR, while Narsingdi was the worst-performing district.

Like the knowledge theme, many demographic and socioeconomic variables were significantly associated with the attitudes towards AMU and AMR. The analysis showed that, when the respondents’ ages increased, the level of favorable attitudes increased. More specifically, the farmers’ aged between 41 and 45 years had 0.11 times, and more than 46 years of age had 3.21 times, favorable attitudes towards AMU and AMR (compared to the farmers aged 18–35 years (OR = 0.11, CI = 0.04–0.32, *p* = 0.000 and OR = 3.21, CI = 1.46–7.05, *p* = 0.004, respectively). The analysis further revealed that the farmers who belonged to high-income groups had 14.30 times more favorable attitudes towards AMU and AMR compared to the middle-income group (OR = 14.30, CI = 4.22–48.44 *p* = 0.000).

The level of education was also found to be positively associated with a favorable attitude. The farmers who completed graduation had 2.49 times more favorable attitudes than the respondents who received education up to the 12th grade (OR = 2.49, 1, CI = 0.39–4.45, *p* = 0.002). Further, the respondents who were raising layer poultry had 0.46 times more favorable attitudes towards AMU and AMR compared to their counterparts, broiler farmers (OR = 0.46, CI = 0.27–0.80, *p* = 0.006). In addition, the farm size was significantly associated with the farmers’ levels of knowledge. The farmers who owned medium-sized farms had 6.96 times more favorable attitudes than those who owned small-sized farms (OR = 6.96, CI = 3.50–13.82, *p* = 0.000). The Chattogram district was one of the best-performing geographic areas in terms of having favorable attitudes towards AMU and AMR compared to Cumilla.

The investigation further revealed that the ages of farmers were associated with the practice of AMU. Specifically, farmers between 36 and 40 years of age performed good practices (OR = 0.24, CI = 0.10–0.59, *p* = 0.002). It was also found that the respondents’ years of experience in poultry farming were associated with the level of good practice. The farmers who had experience between 5 and 8 years, had 7.23 times, 9–12 years had 4.63 times, and 13 or more years had 3.76 times more good practices compared to the farmers with 0–4 years of experience (OR = 7.23, CI = 3.30–15.85, *p* = 0.000; OR = 4.63, CI = 2.08–10.32, *p* = 0.000; and OR = 3.76, CI = 1.30–10.87, *p* = 0.014, respectively). The high-income group of farmers performed 0.35 times more good practices than the middle-income group (OR = 0.35, CI = 0.13–0.89, *p* = 0.028).

Like the knowledge and attitude themes, the level of education found to be a significant association with practice. The farmers who completed graduation performed 2.97 times more good practices related to AMU and AMR compared to those who received education up to the 12th grade (OR = 2.97, CI = 1.61–5.48, *p* = 0.001). The larger-sized farms had 0.36 times more good practices compared to farms with small sizes (OR = 0.36, CI = 0.15–0.88, *p* = 0.024). In terms of the geographic distribution of the good practices related to AMU and AMR, Mymensingh was the best-performing district compared to the other areas (OR = 4.56, CI = 1.78–11.71, *p* = 0.002). 

### 2.7. Relationship between Knowledge, Attitudes, and Practices of AMU and AMR

A Spearman’s rank-order correlation revealed a positive association between each pair of respondents’ knowledge, attitude, and practice scores (*p* ≤ 0.001). The correlations were fair between knowledge–attitudes and knowledge–practices [31] (Table 7).

## 3. Discussion

Over the last two decades, the use of antimicrobials (AMU) and antimicrobial resistance (AMR) has become a burgeoning issue across the world, threatening human and animal health [32,33]. AMR is associated with many factors, such as the misuse and irrational use of antimicrobials, incomplete courses of drugs, and lack of knowledge of antimicrobial use. The reduction of AMR in both public and animal health sectors requires intervention from all stockholders, including farmers who are considered as end users. In the current study, we assessed the knowledge, attitude, and practices (KAP) of layer and broiler poultry farmers towards AMU and AMR. We demonstrated that the respondents’ age, years of experience in farming, level of education, socioeconomic status, and farm type and size influenced the KAP towards AMU and AMR. The findings of the current investigation provide baseline evidence about the KAP of the poultry farmers from low-income resources and offer insights in designing interventions and policy-making of the county.

This study demonstrated that several antimicrobials were used, either alone or combined with other antimicrobials, by broiler and layer farmers to treat different poultry diseases. The government of Bangladesh has banned the use of antimicrobials, including colistin in animals’ feed, for the production of safe animal products by enacting the Fish and Animal Feed Act 2010 [34,35]. However, poultry farmers usually use such antibiotics with drinking water that is used for their poultry. The most commonly used antimicrobials for respiratory diseases such as Newcastle disease and Gumboro were ciprofloxacin, amoxicillin, and tetracycline. These findings were similar to prior studies [27,36], showing the use of several antimicrobials in treating different diseases of broiler and layers birds. One of the key drivers of growing AMR is the misuse of antibiotics associated with the knowledge gap on antimicrobials [36,37], which was also demonstrated during our investigation. Farmers most frequently receive antimicrobials from feed sellers and drug sellers or antibiotic suppliers [36,38]. Usually, they closely work with the representatives of drug companies to achieve their target sales, which may further influence the behaviors of the farmers [38].

Further, small-scale poultry farmers are primarily dependent on credit from poultry sellers [38]. As a result, farmers have to use antibiotics willingly or unwillingly as suggested by the sellers. If feed sellers and drug sellers have a knowledge gap in AMU and AMR, ultimately, those gaps will be reflected in farmers’ behaviors as the end users of antimicrobials. This has also been demonstrated by our recent research [38]. That study found that farmers’ level of education, understanding the facts of antimicrobials, and less exposure to relevant training in drugs and awareness programs in comparison to other stakeholders (e.g., feed and drug sellers), may further explain this situation. In this investigation, it was also observed that a good proportion of poultry farmers did not seek antimicrobials from registered veterinarians, not even for post-mortem examinations of their birds. For doing so, they either depended on other sources of suggestions (such as from drug and feed sellers) or themselves, including the manipulation of doses, duration, frequent switching to antimicrobials if the symptoms did not disappear, and post-mortem examinations. Not seeking registered vets could be due to not much knowing about the services or avoiding the costs related to veterinary services [36,39]. The reasons for not seeking prescriptions from registered vets may have multifaced challenges for farmers. These include the remote locations of farms, difficult access to veterinary services, including laboratory tests for the confirmation of diseases, offering of unskilled services from feed and drug sellers, or the sharing ideas and experiences from neighboring farmers [36,37,39,40]. Another reason could be that farmers can easily buy antibiotics without a prescription in many countries [41,42], including Bangladesh [36]. It has been found that farmers bought antimicrobials most frequently (sole source or in combination with other sources) from feed and chick traders, veterinary medical stores, and even some farmers themselves sell antibiotics [36]. The lack of knowledge of on-farm management, including biosecurity measures, is considered one of the main reasons for the frequent misuse of antimicrobials in poultry farms. Farmers also use antibiotics to compensate for substandard farm conditions, preventing frequently occurring poultry diseases, and as a growth promoter to increase production, resulting in developing AMR [11,43].

The knowledge gap in AMR development originating from poultry industries within resource-limited settings has been extensively discussed [43,44,45]. A recent study demonstrated that the KAP significantly varied by different demographic factors, such as age, years of experience in farming, and level of education of the respondents, including the disease dynamics of the farm and source of information [25]. The findings of the current study were in line with the earlier findings. In particular, the age of the respondents, their years of experience in farming, economic status, and level of education, including farm type and size, were found as the significant predictors that influence the KAP of farmers in the selection and application of antibiotics in the poultry industry.

Like the previous research [32,39,46], the current study demonstrated that the farmers’ age and years of experience in farming are two major significant factors that influence the KAP of AMU and AMR [26]. Specifically, we demonstrated that farmers aged 46 and above with 9–12 years of farming experience positively responded to the KAP of AMU and AMR. Increased age with years of experience may lead to the development of expertise on poultry farming, gaining knowledge for exploring veterinary services and, moreover, exposure to continuous training, awareness programs, and other learning processes of AMU and AMR. Our findings showed a discrepancy with a study [47] that showed that farmers aged 48 years or over were more likely to have a negative attitude towards AMU and AMR. However, the earlier study was unable to show the years of farming experience, which is crucial.

With the above parameters, the level of education of the farmers was another important factor towards the KAP of AMU and AMR. In this study, broiler or layer farmers who completed education up to the graduate level demonstrated positive responses of the KAP related to AMU and AMR. This finding showed consistency with the observations of previous studies [25,26,30,39,48,49]. Better education related to better knowledge is a positive predictor of behavioral changes of the farmers to fight against AMR. Due to a higher level of education, including training and learning processes, farmers may come to know and have more access to facilities of veterinary services, farm management, and biosecurity measures and better understand the use of antimicrobials and their doses’ withdrawal periods [50]. A good level of education and farmers’ behaviors are important in using antimicrobials [25,26]. Like the findings of our study, Ozturk and colleagues demonstrated that postgraduate degree farmers were highly knowledgeable about the use of antibiotics compared to the farmers with high school and primary educations [39]. The farmers with low levels of education (such as a 12th-grade education or below) had to depend on drug sellers and feed sellers, neighboring farmers, and their own experiences, which increased the chances of the misuse of antibiotics and the chance of developing AMR [51].

This study further demonstrated that the knowledge and attitudes of farmers toward AMU and AMR were strongly linked to layer farmers having medium-sized farms compared to their smaller counterparts. Layer farming needs a more extended period to reach the production level, and farmers invest more money to get more benefits. On the contrary, in broiler farming, less time and less investments are required, where farmers get back their investment and profits within a short period. Therefore, those experienced farmers with a good level of education engage more with layer farming compared to broiler farming [52]. We also observed that good practices in AMU and AMR are further associated with larger farm-sized and high-income groups. This is further associated with layer farming, which requires more investment, more experience, and more knowledge, viz. a good level of education [53], as seen in other components of the current study.

Our investigation revealed a correlation between respondents having less knowledge, less appropriate attitudes, and poor practices regarding the AMU and AMR issues. Like other studies of the KAP of AMR [54,55,56], we observed that the level of knowledge influenced the levels of attitudes and practices towards AMU and AMR of farmers. A lower level of knowledge might be associated with the age of the farmers, their level of education, experiences in farming, and economic status. In general, a lower level of knowledge of farmers was due to the lack of training in poultry farming and inadequate consultation with animal care personnel [32]. In the current study, we observed that, for the use of antimicrobials, one-third of the farmers did not seek a prescription from registered veterinarians, instead of relying on other stakeholders or themselves. A proportion of the farmers did not seek post-mortem examinations of the birds to confirm the diseases. The lack of awareness and communication between farmers and veterinarians may lead to the misuse of antimicrobials and be responsible for developing AMR at the farm level [40,57]. Appropriate training and awareness programs on relevant issues, increasing formal or informal education through mass and other media, and the circulation of critical messages through mass and social media can empower farmers’ KAP towards AMU and AMR [27,29,32].

Furthermore, the proper execution of legislations and their strict regulations on drug use, selling, and prescription writing may further improve antimicrobial abuse and help reduce antibiotic resistance [58,59,60]. Increased support by veterinarians and veterinary services may further improve the use of antimicrobials and prevent AMR, as veterinarians are better placed to impact farmers’ behavioral changes [29,32]. They are improving the biosecurity measures also important to avoid the entry of pathogens or dissemination within farms.

This study had several limitations. Firstly, the geographical representation of the sample population. The study included samples from seven districts out of sixty-four districts of Bangladesh. The findings of the current study may not represent the whole. However, the findings of this investigation could provide a comparative picture (as a means of baseline findings) with the other districts of Bangladesh. Secondly, the quality of some of the interview-based data might have been affected by recall bias and self-reported practices amongst the participants. Lastly, although the study assessed the knowledge, attitudes, and practices regarding AMU and AMR, more robust qualitative research is needed to understand the cultural, social, and historical factors to identify the factors associated with KAP differences. Therefore, a qualitative study could be conducted based on the findings of this study.

## 4. Materials and Methods

### 4.1. Study Period and Areas

**Figure 4:**
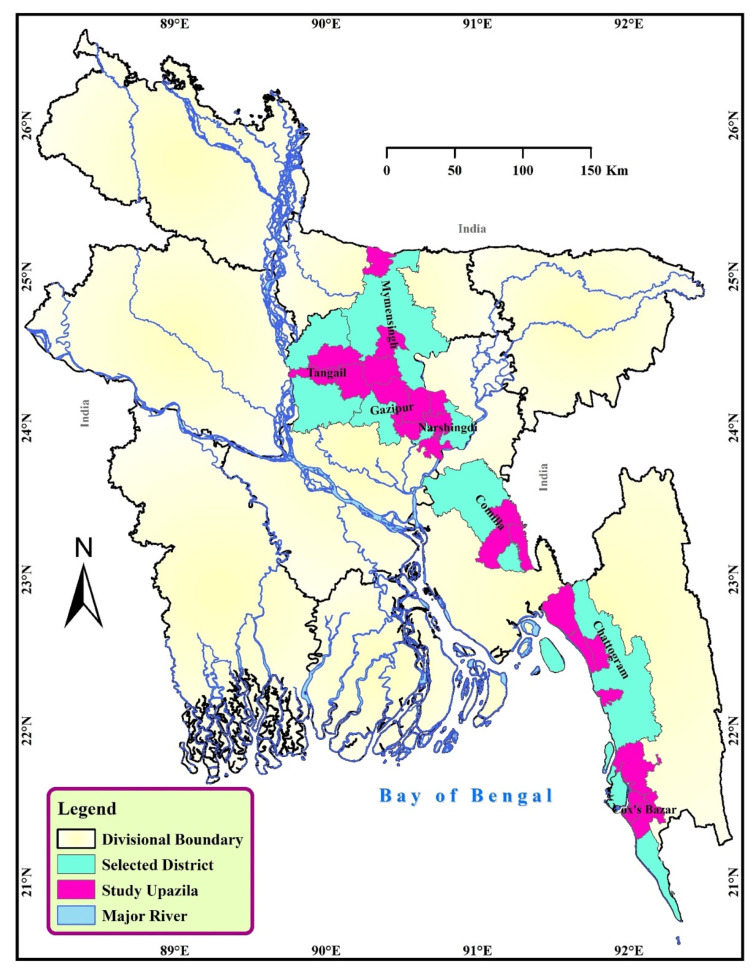
The map shows the study district (color: sea green) and upazilas (color: purple) of Bangladesh.

This study was conducted for six months, commencing from October 2019 to March 2020 in a total of 21 upazilas of 7 districts of Bangladesh—namely, Chattogram, Cumilla, Cox’s Bazar, Gazipur, Mymensingh, Narsingdi, and Tangail (Figure 4). A upazila is considered a subdistrict and the lowest administrative boundary of a district of the country. The study sites were chosen because these areas have a higher number of poultry farms.

### 4.2. Study Design and Sampling

A pre-structured questionnaire on KAP was used to generate cross-sectorial insights, sourcing the farmers involved in chicken meat and egg production in Bangladesh. Farmers were interviewed to elicit their knowledge, attitudes, and self-reported practices regarding AMU and AMR. A single proportion estimation was applied for sample size calculations [61] with a 95% confidence interval, 5% margin of error, and an assumption of that 50% (*p* = 0.5) of poultry farmers used antimicrobials in poultry production, according to the manufacturer’s recommendations. The minimum sample size required was 384 farmers. In the present survey, a total of 420 farmers were interviewed, including 210 layer and 210 broiler farmers from 7 (seven) districts (Chattogram, *n* = 60; Cumilla, *n* = 60; Cox’s Bazaar, *n* = 60; Gazipur, *n* = 60; Mymensingh, *n* = 60; Narsingdi, *n* = 60; and Tangail, *n* = 60) (Table 1). The study sites (districts and upazilas) and farmers (layer and broiler) were selected based on the random sampling method. To select the farmers, we collected a list of farmers from the Upazila Livestock Office. After that, we selected the farmers randomly and approached them to establish if they were available and interested in participating in the study. Once they agreed to take participate in the study voluntarily, the enumerators collected data from them via face-to-face interviews. Those who were not interested or did not have sufficient time and did not agree to give written consent were excluded from the study.

### 4.3. Data Instrument and Collection

The questionnaire included five different sections. In the first section, demographic information such as the age of the farmers (in years), years of experience in farming, economic status, level of education, main occupation, type of farm (broiler or layer poultry raising), farm size, and location were considered (Table 1). The poultry farm sizes were classified as small (up to 4000), medium (4001–10,000), and large (more than 10,000) based on the number of poultry during the data collection [62,63]. The economic status of the farmers was determined by self-reported annual income and categorized by low income (less than USD 1000), middle income (USD 1000–12,500), and high income (more than 12,500). Income data were collected in Bangladeshi currency (BDT) and converted into United States Dollar (USA).

In the second section, the respondents were asked to mention the names of diseases they faced on their farms in the last six months, sources of the antimicrobial they sought for these diseases, whether a post-mortem was performed, and the names of antimicrobials used. The question on recent disease was asked to the respondents initially to mention one major disease name, thereby failing to account for farms that may have had multiple incidents during the six-month period. However, to minimize the recall bias, we recorded one major disease, in terms of severity, and the types of antimicrobials used on that occasion. The third, fourth, and fifth sections consisted of questions related to knowledge (twelve questions), attitudes (nine questions), and practices (eleven questions), respectively. Both negative and positive items were included in each theme. The questionnaire was developed in English primarily and then translated into the local language, Bengali (Supplementary Table S1). The Bengali version was translated back into English and compared with the preliminary version to check the accuracy of the translation. Before collecting the data, the questionnaire was pretested among a few poultry farmers to assess the suitability of the language. Some modifications were made based on the pretesting results to ensure the suitability of the language. The pretested interviews were excluded from the analysis.

### 4.4. Data Management and Analyses

Data obtained from interviews was entered into a paper-based questionnaire and cross-checked. The data was then extracted to an MS Excel spreadsheet for cleaning, processing, and further analysis. We collected data on two categories of closed-ended “yes” and “no” questions on different knowledge, attitudes, and practices related to AMU and AMR. A two-point index (composite score range: 0 to 1) assigned values to responses for knowledge, attitude, and practice items where the correct response (‘yes’) was assigned a value of 1 and the incorrect response (“no”) 0. To analyze how individual participants performed overall in the knowledge, attitude, and practice categories, the sum of each participant’s answers for that particular section was calculated. The data was analyzed using the statistical tool STATA/SE-16.1 (StataCorp, 4905, Lake Way Drive, College Station, TX, USA). Cronbach’s alpha test was used to measure the internal consistency of the themes, with an acceptable value of 0.76 for the knowledge theme, 0.60 for the attitude theme, and 0.63 for the practice theme (0.83 when combining all the themes). We used descriptive statistics, such as frequencies and percentages. Relationships between independent samples were explored using the chi-square test to determine if there were differences among respondents’ characteristics concerning the themes. Using the principal factor method described [64], we identified significant factors in the demographic characteristics and themes. The outcomes regarding knowledge, attitudes, and practices were categorized as “incorrect” vs. “correct”, “unfavorable” vs. “favorable”, and “bad” vs. “good”, respectively. We constructed a summary of these binary outcomes using a two-point index (score less than 1: incorrect/unfavorable/bad and 1: correct/favorable/good).

Furthermore, this factor score analysis was used as a part of the adjusted multivariate logistic regression analysis to determine the association with key themes regarding the respondents’ demographics. Results are expressed as odds ratios (ORs) accompanied by 95% confidence intervals (95% CIs), and a *p*-value < 0.05 was used as the threshold for statistical significance. Spearman’s rank-order correlation coefficient was used to describe the strength and direction of the relationship between responses to the knowledge, attitude, and practice questions.

## 5. Conclusions

Our study of poultry farmers’ knowledge, attitudes, and practices towards AMU and AMR contributes essential information for improving antimicrobials in poultry farms. We demonstrated that the respondents’ socioeconomic demographics, such as education, income source, and age, greatly influence the knowledge, attitudes, and practices of AMU and AMR. The results suggested that one of the key drivers of growing AMR is the misuse of antibiotics associated with the knowledge gap on antimicrobials. Farmers with higher levels of education have more favorable attitudes. In addition, a higher proportion of poultry farmers did not seek a prescribed antimicrobial from registered veterinarians, not even for post-mortem examinations of their birds, which is not good practice. The findings of the current investigation provided baseline evidence about the KAP of poultry farmers from low-income resources and offered insights into designing interventions and policies for the use of antimicrobials in Bangladesh. In particular, the study strongly recommends including farmers as end users of antimicrobials in the policies to combat AMR. Hence, the inclusion of educational and awareness efforts to increase the knowledge, favorable attitudes, and better practices of AMU is highly recommended. 

## Figures and Tables

**Table 1:** Demographic and socioeconomic information of the respondents.

Variables		*n* (%)
Type of farm	Broiler	210 (50.0)
	Layer	210 (50.0)
Farm size	Small (<4000 birds/farm)	262 (62.4)
	Medium (4000–10,000 birds/farm)	99 (23.6)
	Large (>10,000 birds/farm)	59 (14.0)
Age of the farmers (Years)	18–30	140 (33.33)
	31–35	92 (21.9)
	36–40	53 (12.6)
	41–45	51 (12.1)
	46 or more	84 (20.0)
Experience in farming (Years)	0–4	72 (17.4)
	5–8	141 (33.6)
	9–12	153 (36.4)
	13 or more	54 (12.9)
Main occupation	Poultry farming	390 (92.9)
	Other than farming	30 (7.1)
Economic status	Low income (Less than USD 1000/year)	32 (7.6)
	Middle income (USD 1000–12,500/year)	344 (81.9)
	High income (More than USD 12,500/year)	44 (10.5)
Level of education	Graduate	112 (26.7)
	Up to 12th grade	308 (73.3)

**Table 2:** Knowledge of AMU and AMR of broiler and layer farmers.

Items	Total *N* (%)	Broiler Farmers *N* (%)	Layer Farmers *N* (%)	*p*-Value
Do you know who has the authority to write a prescription? (Yes)	360 (85.7)	184 (87.6)	176 (83.8)	0.265
Do you have any idea about antimicrobials? (Yes)	387 (92.1)	185 (88.1)	202 (96.2)	0.002
Do you know about antimicrobial residues? (Yes)	266 (63.3)	112 (53.3)	154 (73.3)	0.000
Do you know about antimicrobial resistance? (Yes)	238 (56.7)	95 (45.2)	143 (68.1)	0.000
Do you know about herbal drugs that can be used as alternatives to antimicrobials? (Yes)	271 (64.5)	108 (51.3)	163 (77.6)	0.000
Do you know any specific antimicrobials that act against a specific disease? (Yes)	355 (84.5)	165 (78.6)	190 (90.5)	0.001
Do you think antimicrobials can be passed to humans through consumption of poultry meat and egg? (Yes)	338 (80.5)	162 (77.1)	176 (83.8)	0.085
Did you know antimicrobials can be used for any type of disease? (No)	299 (71.1)	131(62.4)	168 (80.0)	0.000
Do you think antimicrobials are efficient for the treatment of both bacterial and viral infections? (No)	222 (56.9)	93 (46.7)	124 (59.1)	0.011
Do you think antimicrobials have some side effects? (Yes)	408 (97.1)	199 (94.7)	209 (99.5)	0.003
Do you think the treatment is needed for the whole flock if one/few birds shows any symptoms? (Yes)	383 (91.2)	190 (90.5)	193 (91.9)	0.606
Do you think all antimicrobials can show the same curative effect in poultry diseases? (No)	335 (79.8)	182 (86.7)	153 (72.9)	0.000

**Table 3:** Attitudes towards AMU and AMR in broiler and layer farmers.

Items	Total *N* (%)	Broiler Farmers *N* (%)	Layer Farmers *N* (%)	*p*-Value
Do you believe that the antimicrobials you use randomly might contribute to antimicrobial resistance? (Yes)	261 (62.1)	128 (60.9)	133 (63.3)	0.615
Do you believe that missing a dose may contribute to antibiotic resistance? (Yes)	257 (61.2)	137 (65.2)	120 (57.1)	0.089
Do you think the restriction of antimicrobials can cause more damage than benefits? (Yes)	237 (56.4)	113 (53.8)	124 (59.1)	0.279
Do you think antimicrobials should be added to poultry feed at any time to prevent birds from becoming sick? (No)	264 (62.9)	117 (55.7)	147 (70.0)	0.002
Do you feel the importance of accurate dose of antimicrobials? (Yes)	371 (88.3)	174 (82.7)	197 (93.8)	0.000
Do you think antimicrobials should be placed in restricted areas and accessed only by specific staff when needed? (No)	76 (18.1)	34 (16.2)	42 (20.0)	0.311
When antimicrobials are about to expire, is it better to give medication to the birds to prevent wastage? (No)	376 (89.5)	197 (93.8)	179 (85.2)	0.004
Do you use any herbal or medicinal drugs as alternatives to antimicrobials? (Yes)	301 (71.7)	129 (61.4)	172 (81.9)	0.000
Would you use less antimicrobial, if you knew that the random use of antimicrobials could hamper recovery in the future? (Yes)	338 (80.5)	156 (74.3)	182 (86.7)	0.001

**Table 4:** Practices in AMU and AMR in broiler and layer farmers.

Items	Total *N* (%)	Broiler Farmers *N* (%)	Layer Farmers *N* (%)	*p*-Value
Did you try to use any antimicrobials yourself? (No)	127 (30.2)	40 (19.1)	87 (41.4)	0.000
Do you use any antimicrobials during the brooding period? (No)	76 (18.1)	30 (14.3)	46 (21.9)	0.043
Do you check the expired date before purchasing the drugs? (Yes)	395 (94.1)	190 (90.5)	205 (97.6)	0.002
Do you use antimicrobial as a growth promoter? (No)	238 (56.7)	87 (41.4)	151 (71.9)	0.000
Did you get any suggestions of using antimicrobials from a non-vet? (No)	86 (20.5)	54 (25.7)	32 (15.2)	0.008
Did you get (seek) advice from a vet about the withdrawal period? (Yes)	200 (47.6)	69 (32.9)	131 (62.4)	0.000
Do you maintain an antimicrobial withdrawal period? (Yes)	231 (55.0)	111 (52.9)	120 (57.1)	0.377
Do you increase the dose and frequency of antimicrobials when there are no signs of recovery? (No)	233 (71.1)	96 (45.7)	137 (65.2)	0.000
Do you stop the application of the dose when the birds feel better? (No)	280 (66.7)	119 (56.7)	161 (76.7)	0.000
Do you eat the meat of birds that are given antimicrobials at the end stage? (No)	350 (83.3)	179 (85.2)	171 (81.4)	0.295
Do you shift to using different antimicrobials during the course of a disease? (No)	299 (71.2)	135 (64.3)	164 (78.1)	0.002

**Table 5:** Test of the statistical significances of the variations in the respondents’ knowledge of AMU and AMR by their characteristics.

Variables		Knowledge			Attitudes			Practices		
		Incorrect *n* (%)	Correct *n* (%)	*p*-Value	Unfavorable *n* (%)	Favorable *n* (%)	*p*-Value	Bad *n* (%)	Good *n* (%)	*p*-Value
Age of the farmers (Years)	18–30	65 (46.4)	75 (53.6)	0.000	81 (57.9)	59 (42.1)	0.000	43 (30.7)	97 (69.3)	0.000
	31–35	30 (32.6)	62 (67.4)		49 (53.3)	43 (46.7)		31 (33.7))	61 (66.3)	
	36–40	39 (73.6)	14 (26.4)		28 (52.8)	25 (47.2)		37 (69.8)	16 (30.2)	
	41–45	35 (68.6)	16 (31.4)		41 (80.4)	10 (19.6)		44 (86.3)	7 (13.7)	
	46 or more	45 (53.6)	39 (46.4)		19 (22.6)	65 (77.4)		64 (76.2)	20 (23.8)	
Experience in farming (Years)	0–4	60 (83.3)	12 (16.7)	0.000	41 (56.9)	31 (43.1)	0.000	45 (62.5)	27 (37.5)	0.000
	5–8	58 (41.1)	83 (58.9)		91 (64.5)	50 (35.5)		47 (33.3)	94 (66.7)	
	9–12	62 (40.5)	91 (59.5)		59 (38.6)	94 (61.4)		88 (57.5)	65 (42.5)	
	13 or more	34 (62.9)	34 (37.0)		27 (50.0)	27 (50.0)		39 (72.2)	15 (27.8)	
Economic Status	High income	14 (31.8)	30 (68.2)	0.001	5 (11.4)	39 (88.4)	0.000	34 (77.3)	10 (22.7)	0.000
	Low income	1 (10.0)	9 (90.0)		10 (100.0)	0 (0.0)		1 (10.0)	9 (90.0)	
	Middle income	199 (54.4)	167 (43.6)		203 (51.9)	163 (44.5)		184 (50.3)	182 (49.7)	
Level of Education	Graduate	34 (30.4)	78 (69.6)	0.000	46 (41.1)	66 (58.9)	0.007	42 (37.5)	70 (62.5)	0.000
	Up to 12th grade	180 (58.4)	128 (41.4)		172 (55.8)	136 (44.2)		177 (51.1)	131 (42.5)	
Farm Size	Small	146 (55.7)	116 (44.3))	0.013	172 (65.7)	90 (34.4)	0.000	109 (41.6)	153 (58.4)	0.000
	Medium	38 (38.4)	61 (61.6)		24 (24.2)	75 (75.7)		65 (65.7)	34 (34.3)	
	Large	30 (50.9)	29 (49.1)		22 (37.3)	37 (62.7)		45 (76.3)	14 (23.7)	
Type of Farm	Broiler	130 (61.9)	80 (38.1)	0.000	113 (53.8)	97 (46.2)	0.435	95 (45.2)	115 (54.76)	0.005
	Layer	84 (40.0)	126 (60.0)		105 (50.0)	105 (50.0)		124 (59.1)	86 (40.9)	
Geographic location	Cumilla	38 (63.3)	22 (37.7)	0.005	37 (61.7)	23 (48.3)	0.152	40 (66.7)	20 (33.3)	0.000
	Chattogram	24 (40.00)	36 (60.0)		29 (48.3)	31 (51.7)		25 (41.7)	35 (58.3)	
	Cox’s Bazar	25 (41.7)	35 (58.3)		27 (45.0)	33 (55.0)		26 (43.3)	34 (56.7)	
	Gazipur	38 (63.3)	22 (37.7)		39 (65.0)	21 (35.0)		44 (73.3)	16 (26.7)	
	Mymensingh	28 (46.7)	32 (53.3)		27 (45.0)	33 (55.0)		26 (43.3)	34 (56.7)	
	Narsingdi	37 (61.7)	23 (28.3)		30 (50.0)	30 (50.0)		33 (55.0)	27 (45.0)	
	Tangail	24 (40.0)	36 (60.0)		29 (48.3)	31 (51.7)		25 (41.7)	35 (58.3)	

**Table 6:** Adjusted logistic regression analysis of the factors associated with respondents’ knowledge, attitudes, and practices of AMU and AMR.

Variables		Knowledge	Attitudes	Practices
		OR, 95% CI, *p*	OR, 95% CI, *p*	OR, 95% CI, *p*
Age of the farmers (Years)	18–30	Ref	Ref	Ref
	31–35	1.43, 0.73–2.80, 0.304	1.04, 0.53–2.01, 0.917	0.87,0.43–1.74, 0.686
	36–40	0.13, 0.05–0.34, 0.000	0.70, 0.29–1.69, 0.429	0.24, 0.10–0.59, 0.002
	41–45	0.17, 0.07–0.39, 0.000	0.11, 0.04–0.32, 0.000	0.06, 0.02–0.17, 0.000
	46 or more	0.23, 0.10–0.52, 0.000	3.21, 1.46–7.05, 0.004	0.11, 0.05–0.25, 0.000
Experience in farming (Years)	0–4	Ref	Ref	Ref
	5–8	7.13, 3.16–16.06, 0.000	0.69, 0.34–1.40, 0.301	7.23, 3.30–15.85, 0.000
	9–12	11.54, 4.84–27.51, 0.000	1.63, 0.79–3.37, 0.189	4.63, 2.08–10.32, 0.000
	13 or more	7.27, 2.46–21.45, 0.000	0.94, 0.34–2.57, 0.901	3.76, 1.30–10.87, 0.014
Economic status	Middle income	Ref	Ref	Ref
	High income	2.05, 0.84–4.99, 0.114	14.30, 4.22–48.44, 0.000	0.35, 0.13–0.89, 0.028
	Low income	1.67, 0.62–4.53, 0.313	0.36, 0.12–1.15, 0.085	1.89, 0.60–5.97, 0.279
Level of Education	Up to 12th grade	Ref	Ref	Ref
	Graduate	2.96, 1.69–5.20, 0.000	2.49, 1.39–4.45, 0.002	2.97, 1.61–5.48, 0.001
Type of Farm	Broiler	Ref	Ref	Ref
	Layer	2.01, 1.19–3.39, 0.009	0.46, 0.27–0.80, 0.006	0.62, 0.35–1.11, 0.105
Farm Size	Small	Ref	Ref	Ref
	Medium	3.95, 2.08–7.50, 0.000	6.96, 3.50–13.82, 0.000	0.65, 0.36–1.20, 0.167
	Large	1.03, 0.47–2.24, 0.939	2.45, 1.07–5.60, 0.034	0.36, 0.15–0.88, 0.024
Geographic location	Cumilla	Ref	Ref	Ref
	Chattogram	2.27, 0.88–5.90, 0.091	3.37, 1.20–9.44, 0.021	2.46, 0.89–6.82, 0.084
	Cox’s Bazar	3.20, 1.30–7.87, 0.011	2.33, 0.94–5.79, 0.068	4.07, 1.61–10.29, 0.003
	Gazipur	1.24, 0.51–3.04, 0.635	1.01, 0.40–2.52, 0.991	0.62, 0.24–1.59, 0.319
	Mymensingh	2.77, 1.12–6.83, 0.027	2.73, 1.10–6.80, 0.031	4.56, 1.78–11.71, 0.002
	Narsingdi	1.89, 0.76–4.72, 0.171	1.80, 0.72–4.48, 0.206	3.75, 1.48–9.48, 0.005
	Tangail	2.97, 1.21–7.28, 0.017	2.29, 0.92–5.67, 0.074	3.39, 1.34–8.57, 0.010

**Table 7:** Correlations between knowledge, attitudes, and practices of AMU and AMR.

Variables	Correlation Coefficient	*p*-Value
Knowledge–Attitudes	0.3806	0.000
Knowledge–Practices	0.3472	0.000
Attitudes–Practices	−0.0541	0.2686

## Data Availability

Survey data of knowledge, attitude, and practices on antimicrobial use and antimicrobial resistance among commercial poultry farmers in Bangladesh are given in Table S1.

## Supplementary Materials

The following are available online at https://www.mdpi.com/article/10.3390/antibiotics10070784/s1. Table S1: Survey data of Knowledge, Attitude, and Practices on Antimicrobial Use and Antimicrobial Resistance among Commercial Poultry Farmers in Bangladesh.

## References

[1] DLS (2020). Livestock Economy.

[2] Hamid M., Rahman M., Ahmed S., Hossain K. (2017). Status of poultry industry in Bangladesh and the role of private sector for its development. Asian J. Poult. Sci..

[3] Sarwar M.R., Saqib A., Iftikhar S., Sadiq T. (2018). Knowledge of community pharmacists about antibiotics, and their perceptions and practices regarding antimicrobial stewardship: A cross-sectional study in Punjab, Pakistan. Infect. Drug Resist..

[4] Hassan M.M., Amin K.B., Ahaduzzaman M., Alam M., Faruk M.S., Uddin I. (2014). Antimicrobial resistance pattern against E. coli and Salmonella in layer poultry. Res. J. Vet. Pract..

[5] Hassan M., Ahaduzzaman M., Alam M., Bari M., Amin K., Faruq A. (2015). Antimicrobial resistance pattern against E. coli and Salmonella spp. in environmental effluents. Int. J. Nat. Sci..

[6] Ahaduzzaman M., Hassan M.M., Alam M., Islam S., Uddin I. (2014). Antimicrobial resistance pattern against Staphylococcus aureus in environmental effluents. Res. J. Vet. Pract..

[7] Mahmud T., Hassan M.M., Alam M., Khan M.M., Bari M.S., Islam A. (2016). Prevalence and multidrug-resistant pattern of Salmonella from the eggs and egg-storing trays of retail markets of Bangladesh. Int. J. One Health.

[8] Faruq A., Hassan M.M., Uddin M.M., Rahman M.L., Rakib T.M., Alam M., Islam A. (2016). Prevalence and multidrug resistance pattern of Salmonella isolated from resident wild birds of Bangladesh. Int. J. One Health.

[9] Rahman M., Islam A., Samad M., Islam S., Uddin M., Rumi M., Rostal M., Hagan E., Epistein J., Flora M. (2020). Epidemiological assessment of antimicrobial resistance of Salmonella species from wildlife at human-animal interface in Bangladesh. Int. J. Infect. Dis..

[10] Akter H., Shaikat A., Imtiaz M., Islam A., Khan S., Hassan M. (2019). Prevalence and multidrug resistance pattern of Escherichia coli isolated from street food. Bangladesh J. Vet. Anim. Sci..

[11] Roess A.A., Winch P.J., Ali N.A., Akhter A., Afroz D., El Arifeen S., Darmstadt G.L., Baqui A.H., the Bangladesh PROJAHNMO Study Group (2013). Animal husbandry practices in rural Bangladesh: Potential risk factors for antimicrobial drug resistance and emerging diseases. Am. J. Trop. Med. Hyg..

[12] Chowdhury S., Hassan M.M., Alam M., Sattar S., Bari M.S., Saifuddin A., Hoque M.A. (2015). Antibiotic residues in milk and eggs of commercial and local farms at Chittagong, Bangladesh. Vet. World.

[13] Islam A., Saifuddin A., Al Faruq A., Islam S., Shano S., Alam M., Hassan M.M. (2016). Antimicrobial residues in tissues and eggs of laying hens at Chittagong, Bangladesh. Int. J. One Health.

[14] Sattar S., Hassan M.M., Islam S., Alam M., Al Faruk M.S., Chowdhury S., Saifuddin A. (2014). Antibiotic residues in broiler and layer meat in Chittagong district of Bangladesh. Vet. World.

[15] Khan S.A., Imtiaz M.A., Sayeed M.A., Shaikat A.H., Hassan M.M. (2020). Antimicrobial resistance pattern in domestic animal-wildlife-environmental niche via the food chain to humans with a Bangladesh perspective; a systematic review. BMC Vet. Res..

[16] Hassan M.M., Begum S., Al Faruq A., Alam M., Mahmud T., Islam A. (2018). Multidrug resistant Salmonella isolated from street foods in Chittagong, Bangladesh. Microbiol. Res. J. Int..

[17] Hassan M.M., El Zowalaty M.E., Lundkvist Å., Järhult J.D., Nayem M.R.K., Tanzin A.Z., Badsha M.R., Khan S.A., Ashour H.M. (2021). Residual antimicrobial agents in food originating from animals. Trends Food Sci. Technol..

[18] Uddin M., Ahmed S., Hassan M., Khan S., Mamun M. (2010). Prevalence of poultry diseases at Narsingdi, Bangladesh. Int. J. Biol. Res..

[19] Uddin M., Hossain S., Hasan M., Alam M.N., Debnath M., Begum R., Roy S., Harun-Al-Rashid A., Chowdhury M., Rahman S. (2021). Multidrug Antimicrobial Resistance and Molecular Detection of mcr-1 Gene in Salmonella species Isolated from Chicken. Animals.

[20] Shahana A., Tridip D., Zohorul I.M., Herrero-Fresno A., Biswas P.K., Olsen J.E. (2020). High prevalence of mcr-1-encoded colistin resistance in commensal Escherichia coli from broiler chicken in Bangladesh. Sci. Rep..

[21] Hassan M.M. (2020). Scenario of Antibiotic Resistance in Developing Countries. Antimicrobial Resistance-A One Health Perspective.

[22] World Health Organization (2021). Monitoring Global Progress on Antimicrobial Resistance: Tripartite AMR Country Self-Assessment Survey (TrACSS) 2019–2020: Global Analysis Report.

[23] Ministry of Health and Family Welfare (2017). National Action Plan: Antimicrobial Resistance Containment in Bangladesh 2017–2022.

[24] Boamah V., Agyare C., Odoi H., Dalsgaard A. (2016). Practices and Factors Influencing the Use of Antibiotics in Selected Poultry Farms in Ghana. J. Antimicrob. Agents.

[25] Caudell M.A., Dorado-Garcia A., Eckford S., Creese C., Byarugaba D.K., Afakye K., Chansa-Kabali T., Fasina F.O., Kabali E., Kiambi S. (2020). Towards a bottom-up understanding of antimicrobial use and resistance on the farm: A knowledge, attitudes, and practices survey across livestock systems in five African countries. PLoS ONE.

[26] Kramer T., Jansen L.E., Lipman L.J., Smit L.A., Heederik D.J., Dorado-García A. (2017). Farmers’ knowledge and expectations of antimicrobial use and resistance are strongly related to usage in Dutch livestock sectors. Prev. Vet. Med..

[27] Afakye K., Kiambi S., Koka E., Kabali E., Dorado-Garcia A., Amoah A., Kimani T., Adjei B., Caudell M.A. (2020). The impacts of animal health service providers on antimicrobial use attitudes and practices: An examination of poultry layer farmers in Ghana and Kenya. Antibiotics.

[28] Adekanye U.O., Ekiri A.B., Galipó E., Muhammad A.B., Mateus A., La Ragione R.M., Wakawa A., Armson B., Mijten E., Alafiatayo R. (2020). Knowledge, Attitudes and Practices of Veterinarians Towards Antimicrobial Resistance and Stewardship in Nigeria. Antibiotics.

[29] Rayner A.C., Higham L.E., Gill R., Michalski J.-P., Deakin A. (2019). A survey of free-range egg farmers in the United Kingdom: Knowledge, attitudes and practices surrounding antimicrobial use and resistance. Vet. Anim. Sci..

[30] Pham-Duc P., Cook M.A., Cong-Hong H., Nguyen-Thuy H., Padungtod P., Nguyen-Thi H., Dang-Xuan S. (2019). Knowledge, attitudes and practices of livestock and aquaculture producers regarding antimicrobial use and resistance in Vietnam. PLoS ONE.

[31] Cohen J. (2013). Statistical Power Analysis for the Behavioral Sciences.

[32] Moffo F., Mouiche M.M.M., Kochivi F.L., Dongmo J.B., Djomgang H.K., Tombe P., Mbah C.K., Mapiefou N.P., Mingoas J.-P.K., Awah-Ndukum J. (2020). Knowledge, attitudes, practices and risk perception of rural poultry farmers in Cameroon to antimicrobial use and resistance. Prev. Vet. Med..

[33] Hoque R., Ahmed S.M., Naher N., Islam M.A., Rousham E.K., Islam B.Z., Hassan S. (2020). Tackling antimicrobial resistance in Bangladesh: A scoping review of policy and practice in human, animal and environment sectors. PLoS ONE.

[34] Ministry of Fisheries and Livestock (2010). Fish Feed and Animal Feed Act, 2010.

[35] Goutard F.L., Bordier M., Calba C., Erlacher-Vindel E., Góchez D., de Balogh K., Benigno C., Kalpravidh W., Roger F., Vong S. (2017). Antimicrobial policy interventions in food animal production in South East Asia. BMJ.

[36] Imam T., Gibson J.S., Foysal M., Das S.B., Gupta S.D., Fournié G., Hoque M.A., Henning J. (2020). A cross-sectional study of antimicrobial usage on commercial broiler and layer chicken farms in Bangladesh. Front. Vet. Sci..

[37] Okeke I.N., Laxminarayan R., Bhutta Z.A., Duse A.G., Jenkins P., O’Brien T.F., Pablos-Mendez A., Klugman K.P. (2005). Antimicrobial resistance in developing countries. Part I: Recent trends and current status. Lancet Infect. Dis..

[38] Al Masud A., Rousham E.K., Islam M.A., Alam M.-U., Rahman M., Al Mamun A., Sarker S., Asaduzzaman M., Unicomb L. (2020). Drivers of antibiotic use in poultry production in Bangladesh: Dependencies and dynamics of a patron-client relationship. Front. Vet. Sci..

[39] Ozturk Y., Celik S., Sahin E., Acik M.N., Cetinkaya B. (2019). Assessment of farmers’ knowledge, attitudes and practices on antibiotics and antimicrobial resistance. Animals.

[40] Hasan M.Z.M.G.H., Begum M.R., Kalama M.A., Rahman M.H.F.A.S. (2013). Knowladge, Attitude and Practices of Poultry Farmers about the Use of Antibiotics. Ann. Bangladesh Agric..

[41] Chauhan A.S., George M.S., Chatterjee P., Lindahl J., Grace D., Kakkar M. (2018). The social biography of antibiotic use in smallholder dairy farms in India. Antimicrob. Resist. Infect. Control..

[42] K Landfried L., K Barnidge E., Pithua P., D Lewis R., A Jacoby J., C King C., R Baskin C. (2018). Antibiotic Use on Goat Farms: An Investigation of Knowledge, Attitudes, and Behaviors of Missouri Goat Farmers. Animals.

[43] Khan M., Ferdous J., Ferdous M., Islam M., Rafiq K., Rima U. (2018). Study on indiscriminate use of antibiotics in poultry feed and residues in broilers of Mymensingh city in Bangladesh. Prog. Agric..

[44] Hedman H.D., Vasco K.A., Zhang L. (2020). A Review of Antimicrobial Resistance in Poultry Farming within Low-Resource Settings. Animals.

[45] Glasgow L., Forde M., Brow D., Mahoney C., Fletcher S., Rodrigo S. (2019). Antibiotic use in poultry production in Grenada. Vet. Med. Int..

[46] Alhaji N., Haruna A., Muhammad B., Lawan M., Isola T. (2018). Antimicrobials usage assessments in commercial poultry and local birds in North-central Nigeria: Associated pathways and factors for resistance emergence and spread. Prev. Vet. Med..

[47] Gajdács M., Paulik E., Szabó A. (2020). Knowledge, attitude and practice of community pharmacists regarding antibiotic use and infectious diseases: A cross-sectional survey in Hungary (KAPPhA-HU). Antibiotics.

[48] McNulty C.A., Cookson B.D., Lewis M.A. (2012). Education of healthcare professionals and the public. J. Antimicrob. Chemother..

[49] You J., Yau B., Choi K., Chau C., Huang Q., Lee S. (2008). Public knowledge, attitudes and behavior on antibiotic use: A telephone survey in Hong Kong. Infection.

[50] Ahmed S.M., Naher N., Hossain T., Rawal L.B. (2017). Exploring the status of retail private drug shops in Bangladesh and action points for developing an accredited drug shop model: A facility based cross-sectional study. J. Pharm. Policy Pract..

[51] Kalam M., Alim M., Shano S., Nayem M., Khan R., Badsha M., Mamun M., Al A., Hoque A., Tanzin A.Z. (2021). Knowledge, Attitude, and Practices on Antimicrobial Use and Antimicrobial Resistance among Poultry Drug and Feed Sellers in Bangladesh. Vet. Sci..

[52] Begum I., Buysse J., Alam M.J., Van Huylenbroeck G. (2010). Technical, allocative and economic efficiency of commercial poultry farms in Bangladesh. World’s Poult. Sci. J..

[53] Steen J.T., Ahmad S., Verreynne M.-L., Battese G., Burki A. (2016). Farmers’ Capabilities, Productivity and Profitability: A Case Study of Small Holders in Selected Agro Zones in Pakistan.

[54] Wang Y., Guo F., Wei J., Zhang Y., Liu Z., Huang Y. (2020). Knowledge, attitudes and practices in relation to antimicrobial resistance amongst Chinese public health undergraduates. J. Glob. Antimicrob. Resist..

[55] Waseem H., Ali J., Sarwar F., Khan A., Rehman H.S.U., Choudri M., Arif N., Subhan M., Saleem A.R., Jamal A. (2019). Assessment of knowledge and attitude trends towards antimicrobial resistance (AMR) among the community members, pharmacists/pharmacy owners and physicians in district Sialkot, Pakistan. Antimicrob. Resist. Infect. Control.

[56] Nepal A., Hendrie D., Robinson S., Selvey L.A. (2019). Knowledge, attitudes and practices relating to antibiotic use among community members of the Rupandehi District in Nepal. BMC Public Health.

[57] Reyher K.K., Barrett D.C., Tisdall D.A. (2017). Achieving responsible antimicrobial use: Communicating with farmers. Practice.

[58] Harbarth S., Balkhy H.H., Goossens H., Jarlier V., Kluytmans J., Laxminarayan R., Saam M., Van Belkum A., Pittet D. (2015). Antimicrobial Resistance: One World, One Fight!.

[59] Ouedraogo A., Pierre H.J., Bañuls A., Ouédraogo R., Godreuil S. (2017). Emergence and spread of antibiotic resistance in West Africa: Contributing factors and threat assessment. Med. Sante Trop..

[60] Clifford K., Desai D., da Costa C.P., Meyer H., Klohe K., Winkler A.S., Rahman T., Islam T., Zaman M.H. (2018). Antimicrobial resistance in livestock and poor quality veterinary medicines. Bull. World Health Organ..

[61] Thrusfield M. (2018). Veterinary Epidemiology.

[62] Ennis K.N., Jefferson-Moore K.Y., Bynum J.S. (2008). The Economic Feasibility of Producing Pasture Poultry for Limited Resource Farmers in Southeastern North Carolina.

[63] Center for Integrated Agricultural System (2003). Large-Scale Pastured Poultry Farming in the U.S..

[64] Kim J.-O., Mueller C.W. (1978). Factor Analysis: Statistical Methods and Practical Issues.

